# Mitochondrial MicroRNAs in Aging and Neurodegenerative Diseases

**DOI:** 10.3390/cells9061345

**Published:** 2020-05-28

**Authors:** Albin John, Aaron Kubosumi, P. Hemachandra Reddy

**Affiliations:** 1 Department of Internal Medicine, Texas Tech University Health Sciences Center, Lubbock, TX 79430, USA; albin.john@ttuhsc.edu (A.J.); aaron.kubosumi@ttuhsc.edu (A.K.); 2 Department of Pharmacology and Neuroscience, Texas Tech University Health Sciences Center, Lubbock, TX 79430, USA; 3 Department of Neurology, Texas Tech University Health Sciences Center, Lubbock, TX 79430, USA; 4 Department of Public Health, Graduate School of Biomedical Sciences, Texas Tech University Health Sciences Center, Lubbock, TX 79430, USA; 5 Department of Speech, Language, and Hearing Sciences, Texas Tech University Health Sciences Center, Lubbock, TX 79430, USA

**Keywords:** microRNAs, mitochondrial microRNAs, aging, Alzheimer’s disease, Parkinson’s disease, Huntington’s disease, oxidative stress, mitochondrial function and mitophagy

## Abstract

MicroRNAs (miRNAs) are important regulators of several biological processes, such as cell growth, cell proliferation, embryonic development, tissue differentiation, and apoptosis. Currently, over 2000 mammalian miRNAs have been reported to regulate these biological processes. A subset of microRNAs was found to be localized to human mitochondria (mitomiRs). Through years of research, over 400 mitomiRs have been shown to modulate the translational activity of the mitochondrial genome. While miRNAs have been studied for years, the function of mitomiRs and their role in neurodegenerative pathologies is not known. The purpose of our article is to highlight recent findings that relate mitomiRs to neurodegenerative diseases, including Alzheimer’s, Parkinson’s, and Huntington’s. We also discuss the involvement of mitomiRs in regulating the mitochondrial genome in age-related neurodegenerative diseases.

## 1. Introduction

Since their incorporation into eukaryotes billions of years ago, mitochondria have evolved into the source of energy for cell survival [1]. This energy, in the form of adenosine triphosphate (ATP), powers most cellular processes. Given their origin as independent prokaryotes, mitochondria maintain their own circular DNA separate from somatic DNA [1]. However, the proteins that participate in mitochondrial function come from both nuclear and mitochondrial genomes. This interplay that takes place in the symbiotic relationship between cell and mitochondria results in its peculiar structure and subsequent function [1,2,3,4,5]. Mitochondria perform multiple cellular functions, including ATP production, intracellular calcium regulation, apoptotic cell death, free radicals generation and scavenging, and the activation of the caspase family of proteases in apoptosis [1].

**Figure 1:**
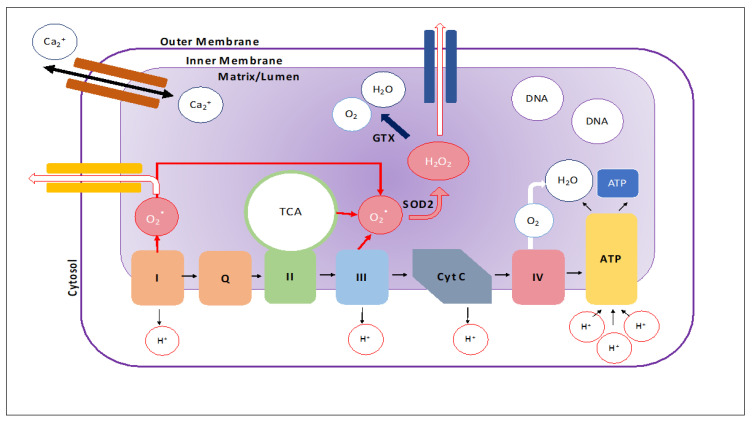
Mitochondrial structure. Schematic of the mitochondrion showing the electron transport chain, flow of ions, and generation reactive oxidative species. These reactive oxidative species can be reduced to hydrogen peroxide via the SOD2 enzyme and further broken down into H_2_O and O_2_ by the GPX enzyme. However, the reduction process is not perfect and when under high stress, reactive oxidative species can leak out of the mitochondria. The mitochondria are also important regulators of intracellular calcium concentration.

A mitochondrion is comprised of an outer membrane, an inner-membrane, and a matrix where its genome, ribosomes, and other structures lie. The intermembrane space is between the outer and inner membranes (Figure 1). The outer membrane is porous and contains proteins, called porins, that allow for the unfettered movement of ions and small, uncharged molecules into the intermembrane space [1,2,3,4,5]. Larger molecules and proteins must pass through translocase complexes to bypass the outer membrane in order to access the intermembrane space. In contrast, the inner membrane serves as a tight barrier around the matrix of the mitochondrion and only allows molecules and proteins access via membrane-transfer proteins that are selective for specific ions and proteins [6]. Invaginations of the inner membrane, called cristae, allow for increased production of ATP by increasing the surface area of the inner membrane [2].

The division between the matrix and intermembrane space allows the mitochondria to perform its most important function of ATP production [2]. The tightness of the inner membrane allows proteins to pump hydrogen ions out of the matrix and into the intermembrane space to create an electrochemical gradient (Figure 1). ATP synthase utilizes this electrochemical gradient to produce ATP in a process called oxidative phosphorylation [1]. In addition to ATP production, the mitochondria play a key role in cell signaling and cellular differentiation. As a result, mitochondrial dysregulation is largely implicated in aging and neurodegenerative diseases [7]. The purpose of this article is to review the latest developments in mitochondrial microRNAs (miRNAs) research in neurodegenerative diseases. This article also focuses on the role of mitochondrial microRNA in neurodegenerative diseases such as Parkinson’s, Alzheimer’s and Huntington’s.

## 2. MicroRNAs

**Figure 2:**
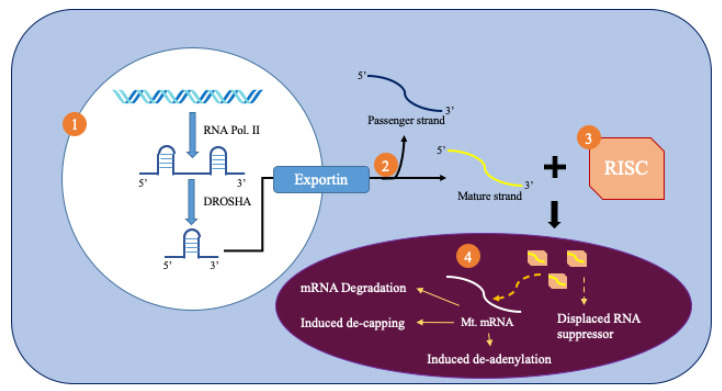
Production and import of mitomiRNA.

MicroRNAs (miRNAs) are small, noncoding RNA molecules that are 18–25 nucleotides in length and act as post-transcriptional regulators of gene expression. Figure 2 summarizes the biogenesis of miRNAs from the nucleus. RNA polymerase II produces primary miRNAs (pri-miRNAs) [8]. These pri-miRNAs are processed inside the nucleus by the drosha protein that produces a precursor miRNA (pre-miRNA) [8]. Pre-miRNA is more stable than pri-miRNA because of its hairpin structure. The pre-miRNA is then exported to the cytoplasm by exportin 5, which recognizes a 2-nucleotide overhang from the hairpin loop. It is then spliced by the dicer RNAse and one strand is chosen due to its greater thermodynamic stability [9]. The chosen strand, or the mature miRNA, binds to the RNA-induced silencing complex (RISC).

MicroRNAs can be found in multiple organelles. Researchers hypothesized that the region near the 3′ end of the miRNA may contain information on intracellular target location. This finding is not absolute as some miRNAs, such as miR-181, can localize in both the cytoplasm (miR-181a) as well as the mitochondria (miR-181c) despite having the same 3′ motif. Regardless, there are several pathways that miRNA may localize to organelles [9].

MicroRNAs perform multiple important functions in the cell, including cell growth, cell proliferation, embryonic development, tissue differentiation, and apoptosis [10]. MicroRNAs have also been found to improve the gene-regulatory processes in cerebrovascular disease. MicroRNA-mediated gene silencing controls a diverse set of important cellular processes, such as cellular senescence and neurodegeneration. Extensive research has also revealed that microRNAs are implicated in a number of human diseases, including cancer, diabetes, obesity, stroke, Alzheimer’s, Parkinson’s, and Huntington’s, as well as aging [8,10,11,12,13]. These circulatory microRNAs can be used as biomarkers for human diseases [8,10,11,12,13].

## 3. Mitochondrial MicroRNAs

Mitochondrial microRNAs (mitomiR), named for their location of regulation, must traverse the controlled double membraned system of the mitochondria to regulate mitochondrial gene expression (Figure 2). It has been hypothesized that Ago2 has a mitochondrial targeting sequence near its amino terminal region that helps direct it towards the mitochondria. As a result, Ago2 levels affect the translocation of miRNA into the mitochondria. Furthermore, impairments of Ago2, such as phosphorylation at particular serine residues, can prevent the miRNA complex from binding with its final site of effect, the mRNA. Pathways, such as KRAS-MEK and ANKRD52-PPP6C, separate Ago2 and its associated miRNA from the RISC complex through the inclusion of P-bodies. These P-bodies can help import miRNA into cellular compartments such as the mitochondria [9].

There are multiple postulations as to how microRNA enter the mitochondria. One such postulation is that Ago2 and the miRNA enter the mitochondria via SAM50 and Tom20 pores (outer membrane) and then through translocation of inner membrane (TIM) [9]. Other proteins, such as GW182 and the PNPase enzyme, may also help with the import of miRNA into the mitochondria. The PNPase enzyme, a 3′ to 5′ exoribonuclease and poly-A polymerase found in the mitochondrial intermembrane space, regulates the entry of miRNA into the mitochondria to maintain homeostasis [14].

Once inside the mitochondrial matrix, miRNA can bind to the 3′UTR of targeted mRNAs produced within the mitochondria and affect mitochondrial gene expression and regulation. This altered gene expression is usually observed as a downregulation. This process can occur through mRNA degradation, reduced ribosome occupancy, induced decapping, induced deadenylation, and altered cap-protein binding. This mRNA degradation has been found to prevent mRNA from reaching translational enzymes [8]. However, in some cases, RISC binding increases gene expression by displacing other stronger RNA repressor proteins.

Although miRNAs have been studied for years, researchers are still scratching the surface when it comes to elucidating the role of mitomiRs in disease and aging pathology. One such burgeoning area of research, for example, is the origin of mitomiRs. Though all mitomiRs observed presently are from nuclear DNA, the mitochondrial DNA can also produce mitomiRs. Mitochondrial DNA is a highly coiled circular DNA that holds 37 genes. While difficult to validate due to a lack of DICER and Drosha enzymes, the mitochondrial DNA may also produce miRNAs from its transcripts. These miRNAs may regulate both within the mitochondria as well as at other organelles such as the nucleus [8,9].

Overall, the science of mitomiRs is emerging and important to know the details of. (1) Nuclear-microRNAs importation into mitochondria; (2) a possibility of mitochondrial genome generated microRNAs; and, most importantly, (3) how mitomiRs regulate mitochondrial gene expression and mitochondrial biogenesis and function. Further research is needed to understand these issues.

## 4. Alzheimer’s Disease

MitomiRs have been associated with Alzheimer’s disease (AD). AD is characterized by a synaptic damage and loss of synapses and an accumulation of amyloid beta and phosphorylated tau in the brain. Such changes alter and destroy synaptic and cognitive functions [15]. Symptoms begin with Mild Cognitive Impairment (MCI) and the inability to form memories. The disease then progresses towards severe cognitive impairment and affects all aspects of intellectual function. The progressive destruction and disruption of synapses culminates in premature cell death. While the complete genesis of the disease is currently unknown, many factors influence its onset and severity.

AD has a profound genetic component. The genes Amyloid Precursor Protein (APP), PSEN1, and PSEN2, which encode proteins involved in APP breakdown and Aβ synthesis, are linked to early onset AD [15]. The APOε4 allele, found on chromosome19q13, is linked to late onset AD in non-Hispanic whites of European ancestry and confers a two- to three-fold risk increase per inherited allele copy [16]. Genome-wide association studies (GWAS) have identified additional genes with varying functions, ranging from APP processing to lipid transportation and immune response, associated with AD pathology [17].

### Mitochondrial microRNAs in Alzheimer’s Disease

MicroRNA play a large role in the pathogenesis and progression of AD [8,11,12]. Most of the miRNAs that have been associated with AD onset are also associated with APP processing [8,18,19,20] and phosphorylated tau [11]. For example, Shu et al. (2018) mimicked the symptoms of AD in mice with the injection of amyloid-β 1-42 and noted a decrease in miR-107 levels. Decreased plasma levels of miR-107 seemed to correlate with the abnormal brain cortical anatomy common to AD patients [21]. The subsequent injection of an miR-107 mimic reversed the induced AD symptoms of spatial memory impairment, decreased phosphorylated tau levels, and decreased Aβ neurotoxicity [21]. MiR-107 affects the oxidative abilities of the mitochondria and reductions in miR-107 can decrease mitochondrial volume and cristae. Furthermore, decreased miR-107 levels can cause mitochondrial dysregulation via a reduction in mitochondrial membrane potential and ETC activity (complexes 1, 3, 4, 5 protein levels decrease) [22].

Li et al. (2017) discovered that miR92a plays an important role in tau-related inducement of anxiety in AD [23]. Tau accumulation in the brain selectively suppresses the expression of the intra-vesicular gamma-aminobutyric acid (GABA) transporter (vGAT) by increasing levels of miR92a, which binds to the vGAT mRNA 3′UTR and inhibits translation. Intervention at any step of the pathway (injection of a GABA antagonist, upregulating vGAT synthesis, or injection of a miR92a inhibitor) showed attenuation of the mice’s anxiety [23].

Other mitomiRs have been implicated in tau hyperphosphorylation and cognitive deficits in AD. Ma et al. (2017) showed that miR-125b was higher in the MCI and AD tissue. Using a terminal deoxynucleotide transferase dUTP nick and end labeling (TUNEL) say, the researchers demonstrated that transfecting cells with miR-125b induced neural cell apoptosis. Western Blot analysis showed that the pro-apoptotic protein Bax and the antiapoptotic protein Bcl-2 were upregulated and downregulated, respectively, suggesting that miR-125b induces cellular apoptosis. Bcl-2 and Bax are proteins found in the cytoplasm that can have a regulatory role in the mitochondrial membrane permeability via the induction of transition pores and the release of cytochrome c. Bcl-2 and Bax work antagonistically such that Bcl-2 suppresses apoptosis while Bax encourages it [24]. This same study showed that miR-125b, when targeting the gene FOXQI, also plays a role in enhancing the expression of phosphorylated tau, which is known to be pathologically related to AD [25].

Weinberg et al. (2015) found evidence of neuronal protection via the downregulation of miR-23a and miR-23b [26]. They performed mRNA expression analysis on brain tissues harvested from subjects with amnestic mild cognitive impairment (aMCI), a phase that precedes AD. They found decreased levels of miR23a and miR23b. Both of these miRNAs are thought to decrease the cellular translation of SIRT1, a protein involved in protective neuronal cell stress responses. Quantitative RT-PCR (qRT-PCR) revealed an upregulation of SIRT1 in the tissue samples of the aMCI subjects relative to the control. In vitro studies confirmed this relation between downregulation of these mRNA and increased expression of SIRT1 [27] leading to inhibition of caspase activation and apoptosis.

Russell et al. (2016) report that oligomeric Aβ42, stimulates TNF-α production, which in turn increases levels of miR-34a, a regulator of phosphorylation complexes I-IV, in the mitochondria [28]. The increased mitochondrial dysfunction results in an increase in cellular APP and an increase in Aβ42. This vicious cycle repeats itself and spreads throughout the neurons of the brain. Shi et al. (2011) demonstrated that miR-743a targets mdh2, malate dehydrogenase, which is an enzyme of the citric acid cycle that is elevated in AD brains [29]. Zhang et al. (2016) showed that miR-195, which is downregulated in AD, partially regulates Mitofusion2 (Mfn2), a protein that plays a role in the fusion of mitochondria [30].

Therefore, there is much evidence that suggests that mitomiRs play a critical role in regulating mitochondrial function under the physiological and pathological conditions of AD. The precise relationship between any abnormal location and distribution of miRNAs in normal functioning/dysfunction of mitochondria in AD still needs to be established. Details of reported miRNAs that regulate mitochondrial functions are given in Table 1. It is possible that an abnormal location and distribution of mitomiRs are involved with impaired mitochondrial dynamics, defective biogenesis, and defective mitophagy. The precise involvement of mitomiRs in these mitochondrial processes in healthy and AD states still needs to be investigated.

## 5. Parkinson’s Disease

Parkinson’s disease (PD) is characterized by resting tremors, muscular rigidity, bradykinesia, and postural inability (citation). Patients with PD do not show any changes in the brain structure unless they also develop dementia. PD-associated dementia results in cerebral atrophy, characterized by the loss of pigmentation in the substantia nigra and locus coeruleus. The loss of dopaminergic neurons in the substantia nigra as well as the formation of Lewy bodies in neurons are the anatomical manifestations of PD. Cell loss is usually found in the lateral ventral tier of the pars-compacta [57]. More than half of the nigral neurons degenerate before symptoms of PD start to show. PD leaves most of its patients with a drastic reduction of up to 80% of their nigral neurons.

Alpha-synuclein is the protein that leads to the formation of Lewy bodies in the neurons of PD patients with dementia. Lewy bodies are spherical inclusions made up of neurofilament proteins and ubiquitin and are around 8 to 30 µm in diameter [58]. Ubiquitin is a cell stress protein that marks damaged or unwanted cellular proteins for degradation [58].

Among all neurodegenerative diseases, PD is most affected by mitochondrial dysfunction. Mitochondrial dysfunction is an early event in PD progression, and mitochondrial abnormalities are involved in almost all PD cases [59]. These abnormalities include: (1) defects in the mitochondrial respiratory chain, (2) defects in mitochondrial DNA, particularly large DNA deletions in nigral cells, (3) increased levels of reactive oxygen species (ROS) (4) calcium dys-homeostasis, particularly in affected nigral cells, (5) defective mitochondrial biogenesis, (6) impaired mitochondrial dynamics (increased fission and reduced fusion), (7) defective mitophagy, and(8) defective axonal transport and distribution of mitochondria in PD neurons [59].

### Mitochondrial MicroRNAs in Parkinson’s Disease

MitomiRs play a key role in the pathogenesis of PD (Table 2). Changes in mitomiR expression trigger mitochondrial abnormalities in mitochondrial activity biogenesis and function. For example, mitomiRs can affect PTEN-induced kinase 1 (PINK1). Cell culture studies revealed that the PINK1-induced impairment of mitochondrial membrane polarization triggers the translocation of parkin from the cytosol to mitochondria [60], promoting the damaged mitochondria to form autophagosomes.

MiR-7 targets Bax and SIRT2, thereby inhibiting the production of pro-apoptotic molecules and reducing neuronal cell death in PD [87]. Mir-7 also downregulates the outer membrane of the protein VDAC1, which aids in maintaining the mitochondrial membrane potential. When stress signals activate VDAC1, VDAC1 opens the pore and destroys the membrane potential, leading to the activation of the apoptotic pathway. The downregulation of VDAC1 by miR-7 increases mitochondrial stability. When miR-7 is downregulated, as is in PD, mitochondrial dysfunction can lead to increased ROS production and the upregulation of VDAC1 [62].

MiR-21 is another microRNA involved in PD pathogenesis and is associated with tumors in PD. In some human tumors, miR-21 levels are significantly increased, which corresponds to the down-regulation of PTEN, a gene that has been found to regulate PINK1, that is involved in the mitophagy pathway [66,88]. Further research is still needed to determine the mechanism by which miRNAs control autophagy and mitophagy in PD.

In PD, the downregulation of miR-34b/c is an early event, but it is not known whether this downregulation triggers mitochondrial dysfunction [70]. Researchers have noted a ballooning of the mitochondria, a decrease in ATP production, and an increase in ROS production [70]. Further research is needed to determine the underlying mechanism by which miR-34b/c misexpression contributes to PD pathogenesis.

MiR-144-3p, downregulated in PD, serves to increase the expression of various genes known to improve the function of mitochondria, including PGC-1α, NRF-1, and TFAM [77]. Several studies have noted an upregulation of miR-155 in mouse models of PD as well as serum samples from PD patients [80,89].

## 6. Huntington’s Disease

Huntington’s disease (HD) is a fatal, genetic neurodegenerative disease that presents with a combination of psychiatric, motor, and cognitive impairments [90] HD is caused by expanded polyglutamine (CAG) repeats in the first exon of the HD gene. The CAG trinucleotide repeats of the HD gene mapped to the p arm of chromosome 4. It is an autosomal dominant disorder that leads to the selective death of striatal neurons and the degeneration of the cerebral cortex that impairs communication between the basal ganglia and cerebral cortex [91]. This disease, named after George Huntington, is most prevalent in Western nations.

Research points to a possible role of the HD gene in the development of the brain, synaptic transmission, vesicle transport, and the regulation of transcription [92]. The expanded CAG repeats a result in a defective gene that can lead to striatal cell death as a result of impaired synaptic transmission of glutamate. However, to compound the defect in glutamate, there is also a defect in GABA release, an important neurotransmitter in the basal ganglia and striatum. Ion influx of calcium and potassium are also impaired in HD [92].

Several cellular changes caused by mutant huntingtin have been reported in HD, including: transcriptional regulation, calcium homeostasis, axonal transport, bioenergetics, oxidative stress, and mitochondrial dysfunction [93,94,95]. Furthermore, mutant huntingtin inhibits the activity of histone acetyltransferase and increases the activity of CREB binding protein (CBP). Mutant huntingtin can also bind to the p53 tumor suppressor protein and increases its activity [91]. PGC-1α, a transcriptional co-activator, is repressed by huntingtin through its stimulation of CREB. PGC-1α is important in regulating mitochondrial gene expression and can activate genes that detoxify reactive oxidative species. The down-regulation of PGC-1α will make the cell vulnerable to increasing oxidative stress. Mitochondrial function is also impaired as a result of defective complex 2. Other complexes of the electron transport chain, such as complex 3 and 4, have also been observed to have diminished activity and function [91].

### Mitochondrial MicroRNAs in Huntington’s Disease

As shown in Table 3, mitomiRs may be involved in regulating mitochondrial dynamics, biogenesis, and mitophagy in HD. However, further research is needed to understand the precise role of mitomiRs in HD.

## 7. Aging

Aging, also considered a neurodegenerative process, is the natural and gradual decline of cellular function. Hallmarks of aging include genomic instability (both nuclear and mitochondrial), telomere attrition, epigenetic alterations, the loss of proteostasis, deregulated nutrient sensing, mitochondrial dysfunction, cellular senescence, stem cell exhaustion, and altered intracellular communication. Cellular senescence, in particular, results from and also drives cellular aging [112]. Senescence is characterized by the induction of the stable arrest of cellular growth and phenotypic changes, including alterations to chromatin remodeling, increased autophagy, the production of an inflammatory cytokine, and increased growth factor of heavy senescence-messaging secrotome. Throughout the lifespan of the cell, various stress factors damage its DNA. The cell initiates senescence to prevent damaged genomes from replicating. Age-induced changes in cellular physiology and structure have been associated with cardiovascular disease, diabetes, cancer, hypertension, and neurodegenerative diseases, such as AD and PD [112].

Mitochondria have been known to be implicated in aging since the 1950s, when the mitochondrial free radical theory of aging (MFRTA) was developed. The reactive oxygen species (ROS) found in the body are primarily produced by mitochondrial damage over time. The accumulation of mitochondrial DNA (mtDNA) damage leads to impaired mitochondrial functionality and cell death. Recent studies cast doubt on the validity of MFRTA, suggesting that neither increased ROS nor upregulating the mitochondrial enzymes that dispose of ROS lead to progeroid syndromes. However, it is clear that mitochondria play an important role in aging and age-related chronic diseases.

**Figure 3:**
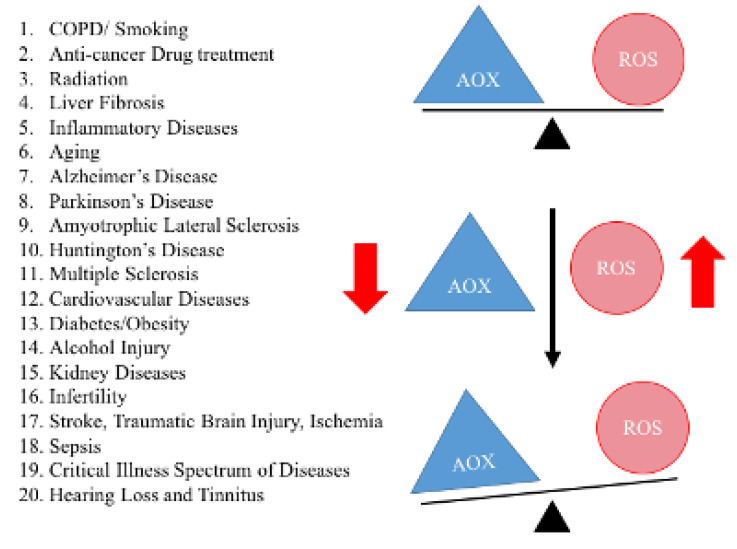
Demonstrates oxidative stress in aging and other age-related diseases. A healthy cell can balance the production of reactive oxidative species and antioxidant enzymes. However, in disease states, such as Alzheimer’s, Parkinson’s, a combination of increased levels of reactive oxidative species production and decreased antioxidant enzymes production have been observed. This imbalance is known as oxidative stress, observed in a large number of human diseases.

Oxidative stress and mitochondrial dysfunction are key players in age-related chronic diseases. As shown in Figure 3, healthy cells can balance the production of ROS with a concomitant production of antioxidant enzymes in the mitochondria. However, under extreme oxidative stress, there may be a combination of increased levels of ROS production and decreased antioxidant enzyme production. The imbalance between ROS production and endogenous antioxidant enzymes, or oxidative stress, is a characteristic feature of the diseases listed in Figure 3.

The increased production of mitochondrial ROS has been hypothesized to be responsible for mitochondrial DNA (mtDNA) mutations. Deletions and single nucleotide changes can reduce mitochondrial function and ATP production. These changes occur cyclically: increased ROS induces changes and ultimately reduces ATP, and vice versa.

To determine other roles of mtDNA in mice, researchers produced mtDNA mutator mice [113], which possess a proofreading-deficient version of the mtDNA polymerase. The mutator mice allow more specific studies of mitochondria and mtDNA damage [113]. Findings from these mutator mice suggest that, in the mitochondria of stem cells, mtDNA mutations alter oxidative phosphorylation and ROS production.

Aging phenotypes have also been associated with aberrant mitochondrial biogenesis that results from signals dependent on mitochondrial metabolism, particularly the insulin/IGF-1 and target of rapamycin pathways nutrient-sensing pathways [113]. The inhibition of these pathways has been shown to extend the lifespan of worms, flies, and some mammals [2]. Reduced nutrient availability has shown the same life-extending properties, although by an unknown mechanism.

MicroRNAs play a role in affecting the mitochondria’s function in aging. Mitochondria in skeletal muscles show aged phenotypes, such as low mitochondrial mass and impaired tolerance to exercise. They present with low levels of miR-133a [114]. MiR-133a interacts with the mitochondrial protein Nix during the regulation of mitochondrial function by controlling mitochondrial membrane potential in muscles. While low levels of mir-133a may cause exercise intolerance, high levels of mir-133a are also deleterious and are seen in heart failure patients [115]. Furthermore, miR-133a is upregulated in aged internal anal sphincter muscles, which has been found to restore tonicity to the muscle fibers [116].

MiR-181a is upregulated in aging dermal fibroblasts and plays a role in extracellular matrix remodeling and tissue senescence. MiR-181a also seems to play a role in age-associated decline in pancreatic function, being under expressed in the β-cells of aged rats [117]. MiR-181a can target NRF1, COX11, COQ10B and other genes by silencing them. The silencing of these genes reduces mitochondrial biogenesis; it also regulates mitophagy via Atg5 and Park2 [118].

Another example of mitomiR misexpression is that of miR-193. miR-193b is upregulated in aging cartilage [119]. Cells with inhibited miR-193b expression showed increased proliferation, suggesting miR-193b involvement in chondrocyte senescence. Furthermore, it can affect the E2F6 pathway, and SOX5 and SOX9 expression [120]. SOX5 can affect mitochondrial membrane permeability via its activity with Bim, Bax and caspases 9 and 3 [121].

## 8. Conclusions and Future Directions

We present recent findings on mitomiRs in aging and age-related neurodegenerative diseases, with a particular emphasis on AD, PD, and HD. MitomiRs have been shown to regulate the translational activity of the nuclear and mitochondrial genomes. Overall, the science of mitomiRs is emerging and may improve our understanding of mitochondrial genome regulation, particularly in disease states such as AD, PD and HD. Current research suggests that over 400 microRNAs are localized to mitochondria and may be involved in mitochondrial genome regulation. However, several important issues need to be addressed: (1) the details of how nuclear-microRNAs import into mitochondria; (2) the details of how mitomiRs regulate mitochondrial gene expression and mitochondrial biogenesis and mitochondrial function; (3) the precise role of each mitomiRs in AD, PD, HD and other neurodegenerative diseases; (4) clarification on whether mitomiRs individually and/or collectively regulate mitochondrial function in all neurodegenerative diseases. Addressing these issues may offer novel solutions to protect against mitochondrial dysfunction and to have more precise control over the transcription of protein products. Further research is still needed to better understand the details of mitomiRs and their role in mitochondrial biogenesis, function and mitophagy.

## Figures and Tables

**Table 1:** Mitochondrial microRNAs related to Alzheimer’s disease.

miRNA	Change	Potential Role	Reference(s)
miR-15a	Upregulation	Targets BACE1	Sørensen et al. (2016) [31]
		increases mitochondrial dysfunction and unbalances mitochondrial membrane potential	Li et al. (2012) [32]
miR-23a/23b	Downregulation	Promote SIRT1	Weinberg et al. (2015) [26]
		Increased SIRT1 affects mitochondrial biogenesis and turnover.	Tang (2016) [33]
miR-27a	Upregulation	Targets the TGF-β pathway	Romano et al. (2017) [34]
		Negatively regulates PINK1 mediated mitochondrial clearance	Kim et al. (2016) [35]
miR-28-3p	Upregulation	APP/PS1 mice	Hong et al. (2017) [36]
		Inhibition of Aldehyde dehydrogenase 2	Li et al. (2015) [37]
miR-34a	Upregulation	Targets ADAM10, NMDAR 2B, and SIRT1	Sarkar et al. (2019) [38]
		Localizes in the mitochondria and downregulates Bcl-2 which increases casp-1 activity. Activates casp-3. Promotes apoptosis and dysfunction of mitochondria	Giuliani et al. (2018) [39]
miR-101	Upregulation	Transcription regulation	Chen et al. (2018) [40]
		Targets STMN1, RAB5A and ATG4D. Inhibits autophagy thus persistence of damaged mitochondria.	Frankel et al. (2011) [41]
miR-107	Downregulation	Reduced expression in the hippocampus	Shu et al. (2018) [21]
		Decreased mitochondrial ETC function and morphological changes	Rech et al. (2019) [22]
miR-125b	Upregulation	Increased Bax, decreased Bcl-2	Ma et al. (2017) [25]
miR-126	Upregulation	GF/PI3K/AKT and ERK signaling cascades	Kim et al. (2016) [42]
		Inhibits complex 1 of mitochondria and reduces aerobic respiration.	Tomasetti et al. (2014) [43]
miR-132	Downregulation	Inhibits complex 1 of mitochondria and reduces aerobic respiration.	Weinberg et al. (2015) [26]
miR-140	Upregulation	ADAM10	Akhter et al. (2018) [44]
		Promotes mitochondrial fission via Mfn1	Duarte et al. (2014) [45]
miR-143	Downregulation	Increased activation of TGF-**β**	Zhang and Wang (2019) [46]
		Increased mitochondrial death as decreased ERK5 pathway	Li et al. (2012) [32]
miR-146a	Upregulation	Associated with mTOR, TNF α	Romano et al. (2017) [34]
		Modulates Bcl-2	Rippo et al. (2014) [47]
miR-155	Upregulation	Associated with mTOR, TNF α	Romano et al. (2017) [34]
		Localizes in the mitochondria	Wang and Springer (2015) [48]
miR-181a	Upregulation	Upregulation of GluA2	Rodriguez-Ortiz et al. (2020) [49]
		Localizes in the mitochondria and downregulates Bcl-2 which increases casp-1 activity. Activates casp-3. Promotes apoptosis and dysfunction of mitochondria	Giuliani et al. (2018) [39]
miR-181c	Upregulation	Down-regulates Bcl-2 and leads to apoptosis	Fisichella et al. (2016) [24]
miR-195	Downregulation	Reduced targeting of BACE1 leads to an increased A**β** levels.	Zhu et al. (2012) [50]
		Reduced mitochondrial ATP production	Yan et al. (2019) [51]
miR-210-3p	Upregulation	Clinical marker for MCI and AD	Siedlecki-Wullich et al. (2019) [52]
		Targets mitochondrial iron sulfur cluster homologue. Decreasing these clusters can reduce activity of mitochondrial enzymes that require iron sulfur clusters. miR-210 can affect aconitase.	Li et al. (2012) [32]
miR-212	Downregulation	Increase SIRT1 in aMCI in the frontal cortex. While it may be protective, sustained downregulation can lead to FOX03a mediated apoptosis.	Weinberg et al. (2015) [26]
miR-330	Downregulation	Affects VAV1 and affects mitochondria through MAPK signaling	Zhou et al. (2018) [53]
miR-424	Upregulation	Cortex white matter	Wang et al. (2011) [54]
		Suppression of ATP levels and mitochondrial integrity through ADP-ribosylation factor-like 2 mRNA.	Duarte et al. (2014) [45]
miR-425	Upregulation	BACE1 protein inhibition	Ren et al. (2016) [55]
		Via RIPK1 causes mitochondrial dysfunction and increased ROS production. Involved in necroptosis.	Hu et al. (2019) [56]

**Table 2:** Mitochondrial microRNAs related to Parkinson’s disease.

miRNA	Change	Potential Role	References
miR-7	Downregulation	Increased a-SYN/Substantia Nigra	Junn et al. (2009) [61]
		Reduced binding to 3′UTR of VDAC1 thus upregulation of anion channel and increased ROS production.	Chaudhuri et al. (2016) [62]
miR-16-1	Upregulation	Decrease HSP70 leading to an increased a-SYN	Zhang et al. (2014) [63]
		HSP70 blocks mitochondrial translocation of Bax, membrane permeabilization, and apoptosis.	Radons (2016) [64]
miR-21	Upregulation	Upregulated in midbrain and directly targets 3′UTR of LAMP2A.	Martinez and Peplow (2017) [65]
		Downregulates PTEN and PINK1, key regulators of mitophagy.	Zhang et al. (2010) [66]
miR-27a/b	Unknown	Increased accumulation and decreased suppression of PINK1 at 3′UTR. Decreased degradation of damaged mitochondria.	Kim et al. (2016) [35]
miR-29a	Upregulation	Mitochondrial voltage dependent anion channel	Lungu et al. (2013) [67]
miR-29b	Upregulation	Loss of mitochondrial membrane potential	Lungu et al. (2013) [67]
miR-30e	Downregulation	Reduced suppression of Nlrp3	Li et al. (2018) [68]
		NLRP3 resides in ER but upon stimulation can interact with mitochondria and cause loss of mitochondrial membrane potential, increase ROS production, and calcium dys-homeostasis.	Liu (2018) [69]
miR-34b/c	Downregulation	Decreased Parkin/DJ-1 leads to increased ROS production in the mitochondria. Decreased ability to reduce MTT. Ballooning of the mitochondria with decreased ATP production.	Miñones-Moyano et al. (2011) [70]
miR-124	Upregulated	Reduced translocation of Bax into mitochondria due to inhibition of Bim.	Wang et al. (2016) [71]
miR-126	Upregulated	Downregulation of IGF-1/PI3K signaling. It targets TOM1, p85beta, insulin receptor substrate 1, CRK	Kim et al. (2014) [72]
miR-137	Slightly Upregulated	Involved in expression of mitophagy receptors FUNDC1 and NIX. Inhibits mitophagy	Li et al. (2014) [73]
			Li et al. (2017) [74]
miR138-2-3p	Downregulation	Increased LRRK2 (lysosomal function in astrocytes)	Cardo et al. (2014) [75]
		LRRK2 localizes in mitochondria and has regulatory function on mitochondrial fission and fusion. Mutations of LRRK2 leads to increased oxidative stress.	Singh et al. (2019) [76]
miR-144-3p	Downregulation	Besides inhibiting the expression of APP, miR-144-3p is involved in the mitochondrial gene expression of PGC-1α, NRF-1, TFAM.	Li et al. (2016) [77]
miR-153	Downregulation	Decreased mTOR signaling	Doxakis et al. (2010) [78]
		Decreased mTOR signaling can lead to reduced clearance of dysfunctional mitochondria (ROS producing) and reduces mitochondrial biogenesis.	Weichhart (2018) [79]
miR-155	Upregulation	Suppression of SOCS-1 and SOC-3 (anti-inflammatory molecules)	Caggiu et al. (2018) [80]
		SOCS-1 suppresses damage to mitochondrial membrane and oxidative stress.	Du et al. (2017) [81]
miR-205	Downregulation	Increased LRRK2	Cho et al. (2013) [82]
		LRRK2 localizes in mitochondria and has regulatory function on mitochondrial fission and fusion. Mutations of LRRK2 leads to increased oxidative stress.	Singh et al. (2019) [76]
miR-494	Upregulation	Decreased DJ-1 (mitochondrial damage)	Xiong et al. (2014) [83]
miR-433	Binding inhibited	Increasing FGF20 (cell death)	Wang et al. (2008) [84]
		FGF20 increases translation of alpha-synuclein, which in turn disrupts calcium exchange between mitochondria and ER. There is a reduction in mitochondrial ATP production.	Paillusson et al. (2017) [85]
miR-4639-5p	Upregulation	Decreased DJ-1 leads to mitochondrial fragmentation	Chen et al. (2017) [86]

**Table 3:** MicroRNAs and their role in Huntington’s disease.

miRNA	Change	Potential Role	References
miR-10b-5p	Upregulation	Targets HOXD10, NF1, KLF4.	Hoss et al. (2015) [96]
		Overexpression of KLF4 can lead to increased ROS production in mitochondria in already impaired mitochondria. KLF4 can also induce mitochondrial fusion.	Wang et al. (2018) [97]
miR-22	Downregulation	Reduced caspase activation and targets HDAC4, Redd1, Rcor1, and Rgs2.	Jovicic et al. (2013) [27]
		Increased Bcl-xl expression leads to decreased pro-apoptotic proteins thus possibly allowing damaged mitochondria to survive.	Liu et al. (2019) [98]
miR-29c	Downregulation	Normally upregulates p53 levels by suppressing p85 alpha.	Lee et al. (2011) [99]
		Reduced levels of p53 may not lead to apoptotic events of damaged mitochondria that have increased ROS production.	Holley and Clair (2009) [100]
miR-124	Downregulation	Decreased PGC-1**α**	Liu et al. (2015) [101]
		Decreased mitochondrial biogenesis and dysfunction (increased ROS, decreased ATP synthesis)	Jin and Johnson (2010) [102]
miR-128a	Downregulation	Tumor repression, apoptosis, epileptic seizure repression	Kocerha et al. (2014) [103]
		Targets FADD. Decreased FADD can prevent apoptosis in damaged mitochondria.	Cavalcante et al. (2019) [104]
miR-132	Downregulation	Decreased AGO2 function	Fukuoka et al. (2018) [105]
miR-146a	Downregulation	HTT gene downregulates miR-146a	Ghose et al. (2011) [106]
		Affect IRAK-1 and TRAF-6 which are inflammation mediators.	Rippo et al. (2014) [47]
		TRAF-6 restricts mitochondrial translocation	Zhang et al. (2016) [107]
miR-196a	Upregulation	Increased BDNF and ANX1A. Increased mitochondrial fusion. Decreased PGC-1**α** and CBP.	Kunkanjanawan et al. (2016) [108]
miR-218	Downregulation	Normally downregulates PRKN. However, in diseased state upregulates PRKN. There is increased mitochondrial degradation and mitophagy as a result.	Di Rita et al. (2019) [109]
miR-222-3p	Upregulation	Targets MMP1, PTEN, SOD2, and other targets	Díez-Planelles et al. (2016) [110]
		Increase THBS1 thus increasing mitochondrial calcium level. Reduces mitochondrial membrane	Zhao et al. (2019) [111]
